# Knowledge, attitude and practice of cervical cancer prevention, among women residing in an urban slum in Lagos, South West, Nigeria

**DOI:** 10.11604/pamj.2019.32.130.14432

**Published:** 2019-03-18

**Authors:** Tope Olubodun, Oluwakemi Ololade Odukoya, Mobolanle Rasheedat Balogun

**Affiliations:** 1 Department of Community Health and Primary Care, Lagos University Teaching Hospital, Lagos, Nigeria; 2 Department of Community Health and Primary Care, College of Medicine of the University of Lagos, Idi-Araba, Lagos, Nigeria

**Keywords:** Attitude, cervical cancer, cervical cancer screening, HPV immunization, knowledge, Nigeria, practice, slum

## Abstract

**Introduction:**

cervical cancer is the most common genital tract malignancy among women in Nigeria. Cancer of the cervix is preceded by a curable premalignant stage which can be detected by screening. The disease can also be prevented by Human papillomavirus (HPV) immunization. Women living in slums usually have poor reproductive health knowledge and poor health behaviours. Mostly of low socioeconomic status, these women are at higher risk of cervical cancer. This study assessed the knowledge, attitude and preventive practices towards cervical cancer among women living in an urban slum in Lagos, Nigeria.

**Methods:**

this descriptive cross-sectional study was carried out among 305 women of reproductive age in Idi-Araba, Lagos, Nigeria. Multistage sampling method was used to select respondents. Data was collected using interviewer administered questionnaires. Analysis was done with SPSS 20 software.

**Results:**

only 39 (12.8%) had heard about cervical cancer. Knowledge of cervical cancer, screening and Human papilloma virus (HPV) immunization was poor. Most respondents (64.3%) did not consider themselves at risk for cervical cancer. However, majority (88.9%) were willing to undergo screening and 93.8% were willing to take HPV immunization or recommend the vaccine to a friend/relative. Only 2(0.7%) had done a cervical cancer screening test and none had taken HPV vaccine or immunized their eligible daughters.

**Conclusion:**

there is thus the need for increased awareness creation and health education programs on cervical cancer prevention among such population of women.

## Introduction

Cervical cancer is one of the most common cancers in women [1] and 80% of cases occur in the developing world. It is a leading cause of mortality from cancers among women living in developing countries [2]. In 2012, new cases of cervical cancer were estimated at 528,000 globally and deaths estimated at 266,000 [3]. In sub-Saharan Africa, 34.8 new cases of cervical cancer are diagnosed per 100,000 women annually and 22.5 per 100,000 women die from the disease. In comparison, in North America the figures are much lower: 6.6 per 100,000 women new cases and 2.5 per 100,000 women deaths [4]. According to the Cervical Cancer Global Crisis Card, Nigeria ranks 5^th^ among countries with regards to death count from cervical cancer, after India, China, Brazil and Bangladesh [4]. Figures from the Ibadan Population Based Cancer Registry (IBCR) covering a 2 year period 2009-2010, show that cervical cancer age standardized mortality rate (ASR) was 36.0 per 100,000 [5] which is higher than in most developed countries. Cervical cancer can have very high human, social and economic costs. It has devastating effects and commonly affects women in their prime [6]. Fortunately, there are measures that offer prevention for this cancer that devastates families, which include screening approaches and vaccines that are efficacious in preventing the infections and precancerous changes that can lead to cervical cancer [2]. Cervical cancer screening, tests for precancerous lesions and cancer in women at risk, most of whom have no symptoms [7]. This includes the conventional Papanicolau (Pap) test, liquid based cytology, visual inspection with acetic acid or lugols iodine (VIA or VILI) and Human papiloma virus (HPV) testing for high risk HPV testing [7]. Three types of vaccines against HPV infection are currently available on the market - gardasil, gardasil 9 and cervarix. They protect against high risk HPV types [8]. Women living in urban slums are mostly of low socioeconomic status and this has been shown to be associated with a higher risk of cervical cancer, poor health knowledge and poor access to health services [9]. This study was thus carried out to determine knowledge, attitude and practice of cervical cancer prevention among women living in Idi-Araba, a slum in Lagos, Nigeria.

## Methods

### Study location

Idi-Araba is one of the political wards in Mushin Local government area of Lagos, Nigeria. It is a densely populated slum with residential houses and shops, many of which are substandard and overcrowded. The area is known as a settlement for the Hausa people although the commonest tribe is the Yoruba tribe. There is a tertiary government hospital located in Idi-Araba, which offers cervical cancer screening services and HPV immunization. The approximate population of Idi-Araba is 48,944 and the predominant occupation is trading.

### Study population

The study was a descriptive cross-sectional study carried out among women living in Idi-Araba community. Eligibility criteria was women of reproductive age (15 - 49 years) who had resided in the community for at least 2 years prior to the study.

### Sampling methodology

The sample size of 305 was determined using the Cochran formula for descriptive studies. A multistage sampling method was used to select respondents. The first stage involved selection of twenty streets out of the total number of streets (fifty streets) using simple random sampling. The second stage involved use of systematic sampling to select houses on each of these twenty streets until the desired number of houses was met. Where there was more than one eligible female in a house, the respondent was selected by balloting. Data was collected using pretested, interviewer administered questionnaires. The questionnaires were administered by three trained female research assistants with a minimum of post-secondary school qualification. Data analysis was done with SPSS 20 software. Frequency tables were made for categorical variables.

### Ethical consideration

Approval was obtained from the ethics and research committee of the Lagos University Teaching Hospital prior to commencement of the study.

## Results

Three hundred and five women were interviewed. The mean age was 33.5± 9.0 years. Most of the respondents were married (73.1%). Of those married, 29.6% were in a polygamous relationship. Majority of the respondents were of the Yoruba tribe (54.4%). A higher proportion of respondents had attained secondary education as their highest level of education (54.1%). Only 8.5% had attained tertiary education. Majority of the respondents were semi-skilled (70.8%) and most of the respondents were of the Islamic religion (Table 1).

**Table 1:** socio-demographic characteristics of respondents

Variable (n = 305)	Frequency (n = 305)	Percentage (%)
**Age (years)**		
15-24	57	18.7
25-34	106	34.8
35-44	94	30.8
45-49	48	15.7
**Mean age =33.5+9.0years**		
Marital status		
Single	57	18.7
Married	223	73.1
Divorced/separated	25	8.2
**Type of marriage (n = 223)**		
Monogamous	157	70.4
Polygamous	68	29.6
Ethnicity		
Yoruba	166	54.4
Hausa	60	19.7
Ibo	43	14.1
Others	36	11.8
**Highest level of education**		
No formal	38	12.5
Primary	76	24.9
Secondary	165	54.1
Tertiary	26	8.5
**Occupation**		
Unemployed	64	21.0
Unskilled	12	3.9
Semi-skilled	216	70.8
Skilled	13	4.3
**Religion**		
Islam	167	54.8
Christianity	136	44.6
Traditional religion	2	0.7
Others	0	0.0

### Knowledge of cervical cancer

Most of the respondents (98.7%) had heard about cancer but only 39 (12.8%) had heard of cervical cancer. About 90% did not know any risk factors of cervical cancer. Some of the risk factors mentioned by respondents were early age at first sex (3.6%), multiple sexual partners (2.0%), infection with HPV (2.0%) and use of tobacco (0.7%). Some of the symptoms of cervical cancer mentioned by respondents include: foul smelling vaginal discharge (5.6%), heavy vaginal bleeding (1.7%) and vaginal bleeding after intercourse (0.7%). Majority of respondents did not know of the symptoms of cervical cancer (90.8%), cervical cancer screening (92.1%) and HPV immunization (98.4%). Most of the respondents’ knowledge of cervical cancer came from the media and the hospital (Table 2).

**Table 2:** respondents knowledge of cervical cancer

Heard of Cervical cancer (n = 305)	Frequency	Percentage (%)
Yes	39	12.8
No	263	86.2
Don’t know	3	1.0
Source(s) of information about Cervical cancer (n = 305)*		
Friends / relatives	5	1.6
Media (television, radio, newspaper, magazines)	20	6.6
Hospital	9	3.0
Religious organization	6	2.0
Internet	2	0.7
Community outreach	0	0.0
Book	0	0.0
Formal lecture	0	0.0
Religious organization	6	2.0
Internet	2	0.7
Community outreach	0	0.0
Book	0	0.0
Formal lecture	0	0.0
Knowledge of risk factors of cervical cancer (n = 305)*		
Early age at first sex	11	3.6
Early age at first pregnancy	2	0.6
Having multiple sexual partners	6	2.0
Having a partner with many sexual partners	2	0.7
Having many pregnancies	2	0.7
Use of tobacco	2	0.7
Infection with HPV	6	2.0
Don’t know	276	90.5
Others	6	2.0
Knowledge of symptoms of cervical cancer (n = 305)*		
Foul smelling vaginal discharge	17	5.6
Heavy vaginal bleeding	5	1.7
Vaginal bleeding in between periods	1	0.3
Vaginal bleeding after intercourse	2	0.7
Vaginal bleeding after menopause	0	0.0
Weight loss	2	0.7
Don’t know	277	90.8
Others	6	2.0
**Heard of cervical cancer screening (n=305)**		
Yes	24	7.9
No	281	92.1
Heard about HPV immunization (n = 305)		
Yes	5	1.6
No	300	98.4

* multiple response

### Respondents’ attitude towards cervical cancer

Majority (64.3%) considered themselves not susceptible to cervical cancer and the commonest reason was believe in spiritual protection (60.7%). However, most respondents (88.9%) were willing to undergo cervical cancer screening when asked, but about seventy percent would require the consent of their spouses. Majority (93.8%) were also willing to be immunized or recommend HPV immunization to a friend or relative. Reasons given for not wanting to be immunized were: the vaccine may cause sexually transmitted infections, could have adverse health effects and could encourage promiscuity among young people (Table 3).

**Table 3:** respondents attitude towards cervical cancer

Percieved susceptibility to cervical cancer (n = 305)	Frequency	Percentage (%)
Yes	54	17.7
No	196	64.3
Don’t know	55	18.0
Total	305	100.0
**Reasons for perceived non susceptibility (n = 196)**		
I do not have casual sex	30	15.3
I currently have only one sexual partner	11	5.6
I have only one lifetime sexual partner	10	5.1
I believe I am spiritually protected	119	60.7
I am personally immune	2	1.0
No reason	13	6.6
Others	19	9.7
**Willingness to undergo cervical cancer screening (n = 305)**		
Yes	271	88.9
No	26	8.5
Not sure	8	2.6
Total	305	100.0
**Need for spousal consent to undergo cervical cancer screening (n = 271)**		
Yes	197	72.7
No	28	10.3
Don’t know	59	21.8
**Willingness to take HPV immunization or recommend it to a friend or relative (n = 305)**		
Yes	286	93.8
No	19	6.2
Total	305	100.0
**Reason(s) why you will not want HPV immunization for yourself or your wards (n = 19)**		
Could lead to increase in promiscuity	1	5.3
Could lead to increase in smoking	0	0.0
Could lead to increase in other STIs	5	26.3
Could have adverse health effects	3	15.8
No reason	10	52.6

### Cervical cancer prevention among respondents

Only 2(0.7%) of the women that took part in the study had done a cervical cancer screening test at some time. One did a pap smear in a tertiary institution and the other, visual inspection with acetic acid (VIA) at an outreach. Reasons given for undertaking screening were: health worker request and test was subsidized. Some reasons given for not undertaking screening include: not being aware of screening (91.4%), lack of symptoms (15.9%), not requested by health worker (2.6%). None of the respondents had taken HPV immunization and none who had female children of 9 years or older, had immunized them (Table 4).

**Table 4:** practice of cervical cancer prevention

Done a cervical cancer screening test (n = 305)	Frequency	Percentage (%)
Yes	2	0.7
No	303	99.3
Total	305	100.0
Place where cervical cancer screening was done (n=2)*		
Private laboratory	0	0.0
Private hospital	0	0.0
Primary health center	0	0.0
Secondary health center	0	0.0
Tertiary health centre (teaching hospital)	1	50.0
Outreach	1	50.0
Reason(s) for doing cervical cancer screening (n = 2)*		
It was part of general screening	0	0.0
Doctor requested it	0	0.0
It was free/subsidized	1	50.0
Because I heard about it and felt I should do it	1	50.0
Others	0	0
Reason(s) for not doing cervical cancer screening (n = 303)*		
I was not aware of cervical cancer screening	277	91.4
I was not aware of facilities where services are available	4	1.3
I did not have any symptoms	48	15.9
It is expensive	3	1.0
For fear of bad result	2	0.7
I believe I can never have cervical cancer	5	1.7
No health worker requested for it	8	2.6
I never thought of it	2	0.7
Others	3	1.0
**Ever had HPV immunization (n = 305)**		
Yes	0	0.0
No	305	100.0
**Have a daughter aged 9years and above (n = 305)**		
Yes	79	25.9
No	226	74.1
Total	305	100.0
Immunized daughter (n = 79)		
Yes	0	0.0
No	0	0.0
Total	0	0.0

* multiple response

## Discussion

This study reported low awareness of cervical cancer. Only 12.8% had heard of cervical cancer. Those aware of cervical cancer screening and HPV immunization comprised 7.9% and 1.6% respectively. Knowledge of health issues, cervical cancer prevention inclusive, is commonly poor among women of low resource, which may explain the level of knowledge reported in this study. A study carried out among women in two urban slums: Makoko waterside and Abete in Lagos, found that only 4.2% were aware of cervical cancer [10]. Similarly, another study among women residing at the urban slums of Old Hubli Karnataka, India, showed that only about 7.5% of the respondents had heard about cervical cancer [11]. A study conducted in two urban slums of Mumbai India, reported 37.7 percent were aware of cervical cancer whereas only 3.6 percent of women were aware of pap smear test [12].

A much larger proportion had heard about cervical cancer in a study among mothers in Shomolu local government area of Lagos State Nigeria [13]. Respondents in the Shomolu study were more educated and that could explain the wide disparity in awareness of cervical cancer (79.6%) [13], as compared with this study (12.8%). Higher level of education is known to be associated with better access to health information. A study in Owerri, capital of Imo state, south west of Nigeria, also reported higher awareness of cervical cancer screening of 52.8% [14]. About three – quarter of the respondents (74.5%) in the Owerri study, had attained tertiary education as compared with 8.5%, less than a-tenth, in this study.

A community based study in Bugiri and Mayuge districts, eastern Uganda, reported that 88.2% of the respondents were aware of cervical cancer, the majority having received information from radio (70.2%) and from health facilities (15.1%) [15]. Living in peri-urban areas, urban areas, having a higher monthly income were associated with better knowledge about cervical cancer prevention [15].

Belief in personal susceptibility was low in this study. Only 17.7% considered themselves susceptible to cervical cancer. A similar finding was observed in a hospital based study in Abakaliki, where only 11.1% felt they were at risk for cervical cancer [16]. The commonest reason given for not being susceptible to cervical cancer in this study was believe in spiritual protection (60.7%). Though most women considered themselves not susceptible to cervical cancer, majority (88.9%) were willing to undergo cervical cancer screening and 93.8% were willing to take HPV immunization or recommend the vaccine to a friend or relative. Majority however said they will require the consent of their spouse to be screened. A finding similar to a study in Zaria where many women had not been screened because they needed their husband’s approval [17].

Although uptake of screening is reported to be low in Nigeria, studies from rural areas and slums have reported lower uptake of cervical cancer screening. Only two (0.7%) respondents in this study had done a cervical cancer screening test at some time. Among women interviewed in two slums in Lagos-Makoko waterside and Abete communities, none had been screened or was aware of a screening test for cervical cancer [10]. In a study at rural Okada a community in Edo state, Southern Nigeria, none had been screened for cervical cancer [18]. A low uptake of cervical cancer screening was however, also observed in a study in Olusosun, a commercial and residential area of Lagos where only 5% of the female respondents had undertaken a pap smear [19]. Similarly, in a study in Onitsha, a metropolitan city in Anambra, Southeast Nigeria, only 1.8% of respondents had done a cervical screening test [20].

A survey in Britain reported 91% of women have had a cervical cancer screening test at least once [21]. Uptake of cervical cancer screening varies globally, being higher in developed countries, as compared with less developed ones. Studies carried out in other developing countries also showed low uptake of cervical cancer screening. In a Kenyan study, uptake was 6% [22] and 0.8% in a community based study in Elmina, Ghana [23].

None of the respondents had taken HPV immunization and none with daughters eligible for HPV vaccination, had immunized their daughters. In contrast, in a study among female health care workers in Enugu, about half of the respondents with adolescent daughters had immunized their daughters [24]. This may be as a result of better knowledge of the vaccine as well as access to the services.

## Conclusion

The women in this study had poor knowledge of cervical cancer and majority felt not susceptible to the disease. Uptake of cervical cancer screening and HPV immunization was low. Most however, expressed willingness to undergo screening and be immunized. There is thus, need for increased cervical cancer awareness and promotion campaigns. Women’s partners should also be targeted for health education. Improving access to cervical cancer prevention services is also crucial among this underserved population.

### What is known about this topic

- Cervical cancer is one of the most common cancers in women;
- It is a leading cause of mortality from cancers among women living in developing countries.

### What this study adds

- Low awareness of cervical cancer was reported among the slum dwelling women in this study;
- Belief in personal susceptibility was low but most participants were willing to be screened or vaccinated. However, the majority will require the consent of their spouses;
- This highlights the need for health education campaigns on cervical cancer prevention with involvement of males, as well as increasing access to cervical cancer preventive services among low resource women.

## Competing interests

The authors declare no competing interests.
